# Citrus Leprosis Virus C Encodes Three Proteins With Gene Silencing Suppression Activity

**DOI:** 10.3389/fmicb.2020.01231

**Published:** 2020-06-09

**Authors:** Mikhail Oliveira Leastro, Deibis Yorlenis Ortega Castro, Juliana Freitas-Astúa, Elliot Watanabe Kitajima, Vicente Pallás, Jesús Ángel Sánchez-Navarro

**Affiliations:** ^1^Unidade Laboratorial de Referência em Biologia Molecular Aplicada, Instituto Biológico, São Paulo, Brazil; ^2^Instituto de Biología Molecular y Celular de Plantas, Universidad Politécnica de Valencia-Consejo Superior de Investigaciones Científcas (CSIC), Valencia, Spain; ^3^Embrapa Mandioca e Fruticultura, Cruz das Almas, Brazil; ^4^Departamento de Fitopatologia e Nematologia, Escola Superior de Agricultura Luiz de Queiroz, Universidade de São Paulo, Piracicaba, Brazil

**Keywords:** RNA silencing suppressor, citrus leprosis virus C, RSS activity, hypersensitive response, family *Kitaviridae*

## Abstract

Citrus leprosis virus C (CiLV-C) belongs to the genus *Cilevirus*, family *Kitaviridae*, and is considered the most devastating virus infecting citrus in Brazil, being the main viral pathogen responsible for citrus leprosis (CL), a severe disease that affects citrus orchards in Latin America. Here, proteins encoded by CiLV-C genomic RNA 1 and 2 were screened for potential RNA silencing suppressor (RSS) activity by five methods. Using the GFP-based reporter agroinfiltration assay, we have not found potential local suppressor activity for the five CiLV-C encoded proteins. However, when RSS activity was evaluated using the alfalfa mosaic virus (AMV) system, we found that the p29, p15, and p61 CiLV-C proteins triggered necrosis response and increased the AMV RNA 3 accumulation, suggesting a suppressive functionality. From the analysis of small interfering RNAs (siRNAs) accumulation, we observed that the ectopic expression of the p29, p15, and p61 reduced significantly the accumulation of GFP derived siRNAs. The use of the RSS defective turnip crinkle virus (TCV) system revealed that only the *trans*-expression of the p15 protein restored the cell-to-cell viral movement. Finally, the potato virus X (PVX) system revealed that the expression of p29, p15, and p61 increased the PVX RNA accumulation; in addition, the p29 and p15 enhanced the pathogenicity of PVX resulting in the death of tobacco plants. Furthermore, PVX-p61 infection resulted in a hypersensitive response (HR), suggesting that p61 could also activate a plant defense response mechanism. This is the first report describing the RSS activity for CiLV-C proteins and, moreover, for a member of the family *Kitaviridae*.

## Introduction

Organisms have a primary cellular defense mechanism known as RNA silencing. RNA silencing has a fundamental “sequence specific gene regulatory feature” (Kakumani et al., 2013) and plays an important role in defense against invading microorganisms (pathogens), especially viruses (Pumplin and Voinnet, 2013). This defense mechanism is activated by double-stranded RNA from high genome amplification of invasive microorganisms, transposons or ectopic expressed genes (Aravin et al., 2001; Pumplin and Voinnet, 2013; Guo et al., 2016). Briefly, double-stranded RNAs are processed by dicer-like RNases (DCLs) in small RNAs (siRNAs) of 20–24 nt in size (Hamilton and Baulcombe, 1999; Matranga and Zamore, 2007; Borges and Martienssen, 2015) then the small RNAs are loaded onto Argonaute (AGO) proteins to guide the silencing of DNA or RNA elements by a specific recognition of sequence complementarity (Hammond et al., 2000; Ding and Voinnet, 2007; Pumplin and Voinnet, 2013; Nakanishi, 2016; Pisacane and Halic, 2017). On the other side, in counter-defense against the RNA silencing antiviral defense, viruses evolved to encode suppressors of RNA silencing proteins (RSS) (Voinnet et al., 1999; Roth et al., 2004; Li and Ding, 2006; Burgyan and Havelda, 2011; Csorba et al., 2015; Moon and Park, 2016) which may differ by their ability to suppress intracellular and/or intercellular silencing machinery (Li and Ding, 2001; Lu et al., 2004, 2005; Merai et al., 2006; Garcia and Pallas, 2015; Samuel et al., 2016).

As reviewed by Martinez-Perez et al. (2019), several viral RSSs have been identified using different procedures (Moissiard and Voinnet, 2004; Roth et al., 2004; Qu and Morris, 2005; Li and Ding, 2006; Gupta et al., 2018; Yang et al., 2018; Martinez-Perez et al., 2019). The most common assay is the “patch” technique (Voinnet et al., 1998) a system that uses *Agrobacterium tumefaciens* cultures harboring the putative RSS and a reporter gene infiltrated on *Nicotiana benthamiana* leaves, in which the natural silencing process of the overexpressed reporter gene is delayed by the presence of an RSS. The RSS screening based on viral vector, such as potato X virus (PVX), in which the expression of an RSS is associated with a more aggressive viral infection (Voinnet et al., 1999), is also widely used. Functional complementation of defective viral mutants (Chiba et al., 2006; Powers et al., 2008) or viral vectors in which the RSS is correlated with symptoms appearance (Guilley et al., 2009) have been used to identify RSS in the last decade.

Recently, a new approach to screen for RSS activity based on a viral system derived from alfalfa mosaic virus (AMV) has been presented, which revealed a correlation between the presence of necrotic lesions on inoculated leaves and RSS activity (Martinez-Perez et al., 2019).

Citrus leprosis virus C is the type member of the genus *Cilevirus*, family *Kitaviridae* (Locali-Fabris et al., 2012; Freitas-Astua et al., 2018; Quito-Avila et al., 2020). It is the main viral pathogen responsible for the citrus leprosis, a re-emergent disease that considerably affects citrus production in Latin America. “Its genome is composed of two linear positive sense ssRNA segments with the presence of 5’cap structure and a 3’ poly(A) tail, organized in six open reading frames (ORFs). The first segment (RNA1) has two ORFs that code for a replication-associated protein containing conserved domains of methyl transferase, helicase, and RNA dependent RNA polymerase, and the capsid protein (p29)” (Locali-Fabris et al., 2006; Pascon et al., 2006; Leastro et al., 2018). The second segment (RNA2) encodes four proteins: (i) p15, a small protein for which a specific function needs to be determined, but potentially involved with virus replication as suggested by its involvement in the formation of vesicles thought ER remodeling (Leastro et al., 2018); (ii) p61, which exhibits features of glycoprotein (Kuchibhatla et al., 2014; Leastro et al., 2018) with biological activity on remodeling the ER system and redistributing the Golgi apparatus (Leastro et al., 2018); (iii) p32 which has the function of a movement protein (MP) (Leastro et al., unpublished); and (iv) p24, an integral membrane protein with the ability to form vesicle-like spherical structures in association with the ER, which strongly suggests the involvement of this protein in viral replication and assembly (Leastro et al., 2018). Those features, in addition to membrane topology presented for the p24 protein (Leastro et al., 2018) combined with its homology to virion membrane proteins of plant and arthropod viruses (Kuchibhatla et al., 2014; Leastro et al., 2018) suggest a potential structural role as a matrix protein. The natural infection by citrus leprosis-associated viruses results only in localized lesions in field conditions; for a yet unknown process, these viruses seem to be unable to infect phloem tissues and, therefore, are unable to become systemic in their hosts.

Molecular aspects related to the mechanism of cileviruses infection have been elucidated in recent years; however, some important features remain to be explored. Currently, there is no information about the RSS activity of the proteins encoded by the cileviruses. In this work, we provide a step further in the molecular understanding of how these viruses cause infection. Here, we screened the CiLV-C proteins to identify potential RSS activity by using five systems well-described in the literature. We show that the CiLV-C p29, p15, and p61 proteins have suppressor activity, thus elucidating the functionality of p15, and characterizing additional functions for the capsid protein (p29) and the putative glycoprotein (p61). Furthermore, the p61 expression on the PVX infection context generates a hypersensitive response (HR), suggesting that this protein could also activate a plant defense response mechanism.

## Materials and Methods

### DNA Manipulation

An infectious cDNA 3 construct of AMV that expresses green fluorescent protein-GFP (pGFP/MP/CP) (Sanchez-Navarro et al., 2001) was used to express the potential RSS proteins by exchanging the GFP gene. The introduction of the Human influenza hemagglutinin epitope (HA) at the N- or C-terminus of the GFP was performed as previously described (Martinez-Perez et al., 2019). The resultant pGFP:HA/MP/CP construct expressed the GFP with the HA epitope at the C-terminus and allowed the exchange of the GFP gene by using the *Nco*I and *Nhe*I sites. The CiLV-C genes *p29*, *p15*, *p61*, *MP*, and *p24* (GenBank accessions YP_654539.1, YP_654540.1, YP_655441.1, YP_654542.1, and YP_6545543.1, respectively) were amplified by PCR with specific primers containing the *BspH*I/*Nhe*I (*p29*), *Pci*I/*Nhe*I (*p15* and *p24*), or *Nco*I/*Nhe*I (*p61* and *MP*) restriction sites. The corresponding fragments were inserted into an AMV RNA 3 clone. The insertion of the p61 with the HA at its N-terminus (pHA:p61/MP/CP) was performed as previously described (Martinez-Perez et al., 2019). The specific primers for p61 frame shift amplification generated an amplicon carrying the 5’*Nco*I and 3’*Nhe*I restrict sites plus a stop (TAA) sequence after the fifth codon. This PCR product, previously digested, was cloned into the pGFP:HA/MP/CP construct, as aforementioned, to generate the construction pp61(fs)stop:HA/MP/CP. The AMV construction harboring the RSS gene of tobacco etch virus (TEV) *HCPro* (GenBank accession DQ986288) was obtained from Martinez-Perez et al. (2019).

For transient expression of the proteins, the amplified genes above described carrying stop codons were introduced in the expression cassette of the plasmid pSK35S-MP_TSWV_:HA-PoPit (Leastro et al., 2015) by exchanging of the tomato spotted wilt virus (TSWV) MP. The cassettes resulting were under the control of 35S constitutive promoter from cauliflower mosaic virus (CaMV) and the terminator from the potato proteinase inhibitor (PoPit) (Leastro et al., 2015). Then, the corresponding expression cassettes (35S-ORF_stop_:HA-PoPit) were subcloned into the pMOG_800_ binary vector by using the restriction sites *Hind*III (for *p15* and *p61*) and *Xho*I (for *p29*, *MP*, and *p24*). The leader peptidase (Lep) construct used as negative control was obtained from Peiro et al. (2014).

For the PVX assay, the heterologous viral genes were introduced in a PVX expression vector (Lu et al., 2003). For that, the CiLV-C corresponding genes were amplified by PCR with primers carrying *Sal*I restriction site. The amplicons were digested and cloned into the plasmid pGR107, previously digested with *Sal*I and dephosphorylated. *HCPro* and coat protein (CP) of carnation mottle virus (CarMV) (GenBank acc.: AJ304989) were obtained from Martinez-Perez et al. (2019).

### *N. benthamiana* Wild Type and 16c RNA Silencing Suppression Assay

To study the effect of different viral factors in intracellular and intercellular RNA silencing suppression, we performed the agroinfiltration technique using wild type or GFP transgenic *N. benthamiana* plants (line 16c) (Hamilton et al., 2002; Burgyan and Havelda, 2011). pMOG_800_ binary constructions harboring the *HCPro* and *p29*, *p15*, *p61*, *MP*, and *p24* CiLV-C genes were introduced into C58C1 cells and kept overnight at 28°C in Luria-Bertani (LB) broth with rifampicin and kanamycin. pMOG(GFP) construct carrying the eGFP gene was used to trigger the silencing of the GFP transgene of 16c tobacco plants or to generate small RNAs (siRNAs) in both wt and 16c line of *N. benthamiana*. In co-infiltration experiments, we performed a mixture in equal volume (OD_600_ = 0.5) of *A. tumefaciens* culture containing pMOG-GFP binary plasmid and individual cultures harboring each of the above mentioned viral factors. A mixture consisting of equal volume of *A. tumefaciens* cultures carrying pMOG(GFP) or empty pMOG_800_ were used as controls. Three independent experiments were performed, each one included the infiltration of 10 plants per construct. The plants were grown under two-step cycle of 10 h of darkness at 18°C and 14 h of light at 20°C or under conditions of 23°C day 18°C night and 16 h light/8 h dark regime maintained in FITOTRON^®^ plant chamber. Agroinfiltrated leaves were photographed at 6 days post-inoculation (dpi) under long-wavelength UV light (UVGL-58 Handheld UV lamp; UV Products) by using a tripod and a Nikon D3000 digital camera at F11 aperture value and 1/10 s shutter speed (Yaegashi et al., 2012; Martinez-Perez et al., 2019).

### Alfalfa Mosaic Virus Necrotic Response Assay

Plasmids of the pGFP:HA/MP/CP chimeric AMV RNA 3 constructs harboring the *HCPro*, *p29*, *p15*, *p61*, *MP*, and *p24* viral factors were linearized with *Pst*I and transcribed with T7 RNA polymerase (Takara Bio USA, Inc.) following the manufacturer’s instructions. Transgenic *N. tabacum* plants that express the polymerase proteins P1 and P2 of AMV (P12 plants) (Van Dun et al., 1988) were grown and inoculated with RNA transcripts, as described previously (Taschner et al., 1991). P12 protoplasts were extracted and 2.5 × 10^5^ protoplasts were inoculated by the polyethylene glycol method (Loesch-Fries et al., 1985) with 15 μL of the transcription mixture.

On the surface of the P12 mechanically inoculated leaves, the presence of necrotic lesions was monitored for 2 weeks post-inoculation with onset of lesions observed at 4 dpi. Three independent experiments were performed, each one included the infiltration of three or four leaves from three plants per construct.

### Turnip Crinkle Virus Complementation and Co-infiltration Assays

The turnip crinkle virus (TCV) assay was performed in two different approaches: (i) as an infectious RNA transcript referred to as TCV-sGFP complementation assay or (ii) via agroinfiltration referred to as PZP-TCV-sGFP co-infiltration assay (Powers et al., 2008). In the first method, the movement-deficiency phenotype of a TCV CP deletion mutant that expresses GFP (TCV-sGFP) is complemented in *trans.* In the second approach, the presence of an RSS is identified by an increase of the GFP signal in the whole leaf previously agroinfiltrated with PZP-TCV-sGFP constructs (Martinez-Perez et al., 2019). For the TCV-sGFP complementation assay, three *N. benthamiana* leaves per plant and three plants per construct were agroinfiltrated with *A. tumefaciens* cultures (strain C58) carrying the pMOG-empty or expressing the *Lep*, as negative controls, or the pMOG_800_ constructs expressing *HCPro*, *p29*, *p15*, *p61*, *MP*, and *p24* at OD_600_ = 1 (Powers et al., 2008). Next, the pTCV-sGFP plasmid was linearized with *Xba*I and, 1 day post-infiltration, TCV-sGFP infectious RNA transcripts were mechanically inoculated onto the abaxial surfaces of the infiltrated leaves as referred by Martinez-Perez et al. (2019). Local movement was evaluated at 3 dpi, with the aid of a Leica MZ16F fluorescence stereomicroscope. For the co-infiltration assay, the *A. tumefaciens* cultures aforementioned were mixed with an *Agrobacterium* culture carrying the PZP-TCV-sGFP construct at an OD_600_ = 0.0025 (Powers et al., 2008) and then infiltrated in *N. benthamiana* leaves. The GFP signal of the infiltrated leaves was monitored at 5 dpi with a Leica MZ16F fluorescence stereomicroscope. Each assay was repeated three times by inoculation of three *N. benthamiana* leaves per plant and three plants per construct.

### Potato Virus X Pathogenicity Assay

PVX-derivatives either containing the *HCPro*, CarMV *CP*, *p29*, *p15*, *p61*, *MP*, or *p24* were constructed by cloning each ORF into the *Sma*I (for *HCPro* and CarMV *CP*) or *Sal*I (for CiLV-C ORFs) sites of pGR107 downstream of the duplicated PVX CP promoter (Jones et al., 1999). *N*. *benthamiana* plants were agroinfiltrated (OD_600_ = 0.5) (Martinez-Perez et al., 2019) with *A. tumefaciens* strain C58C1 harboring the helper plasmid pSoup and electroporated with each of the recombinant construct. The empty pGR107 was used as negative control. Total RNA extraction was performed at 4 days post-infiltration from upper non-infiltrated leaves using VWR Life Science AMRESCO RiboZol^TM^ RNA Extraction Reagent following the manufacturer’s instructions. Three independent experiments were performed, each one included the infiltration of three plants per construct. Three weeks later, entire plants were photographed. The symptom development was monitored for up to 30 days post-infiltration.

### Northern Blot Assays

Total RNA was extracted from P12 protoplasts at 16 h post-inoculation and from *N. benthamiana* leaves expressing transiently tested proteins in combination with the TCV (at 5 dpi), PVX (at 4 dpi) and 16c agroinfiltration systems (at 4 dpi), using VWR Life Science AMRESCO RiboZol^TM^ RNA Extraction Reagent. After electrophoresis through formaldehyde-denatured gel, the RNAs were transferred to positively charged nylon membranes (Roche Mannheim, Germany) (Leastro et al., 2017) and fixed with a UV cross-linker (700 × 100 μJ/cm^2^). Hybridization and detection was conducted as previously described (Pallas et al., 1998) using a dig-riboprobe (Roche, Mannheim, Germany) complementary to the 3’UTRs of the AMV RNA 3 and TCV. For detection of the genomic (g) and sub genomic (sg) PVX RNAs, northern blot assays were performed by overnight hybridization with a 500 nt length digoxigenin (DIG)-labeled-riboprobe complementary to the 3’ end region of the PVX *CP* gene.

For the analysis of the small RNAs, total RNA was extracted from 0.1 g of *N. benthamiana* infiltrated leaves at 4 days post-infiltration, using TRI Reagent (Sigma-Aldrich, Steinheim, Germany). Nine micrograms of the total RNAs were electrophoresed through a 17% denaturing polyacrylamide gel and transferred to positively charged nylon membranes (Roche, Mannheim, Germany). RNAs were fixed, hybridized and detected as described above with the difference that the hybridization was performed at 38°C using a mix of three 50 nt dig-riboprobe complementary to nt: 707–756, 761–810, 881–930 of the GFP gene (GenBank accession U76561).

For analysis of mGFP expression, RNAs were extracted at 4 days post-infiltration and hybridized using dig-riboprobe complementary to the complete sequence of the GFP gene.

### Statistical Analysis

Each assay reported here was performed in triplicate unless specifically noted otherwise. Standard deviation (±SD) represents data from three biological samples with at least three replicates for each sample. Student’s *t*-test was performed to determine the significant differences between control specified and viral factor (sample) at each experiment. Significant difference is demonstrated by values lower than *p* < 0.05. ^∗^, ^∗∗^, or n.s represent *p* < 0.05, 0.01, or no significant difference, respectively. The graphs represent the relative accumulation of the RNAs corresponding to the average of three northern blot analyses from three independent experiments. The mean values, obtained from the band quantification, were normalized to the control mean values. The bands were quantified using ImageJ version 2.0cr software with ISAC plugin.

### Western Blot Analysis

P12 leaves inoculated with RNA3 chimeric constructions at 3 days post-inoculation were processed with 250 μL of Laemmli loading buffer 1X (Laemmli, 1970). After boiling for 5 min, 25 μL of the mixture were subjected to 12% SDS-PAGE. Proteins were detected on Western blots using a mouse monoclonal anti-HA antibody (Sigma-Aldrich, Steinheim, Germany) and a secondary anti-mouse peroxidase labeled antibody (Sigma-Aldrich, Steinheim, Germany) together with a chemiluminescence substrate (Amersham^TM^ ECL^TM^ Prime Western Blotting Detection Reagent). The chemiluminescence detection was performed with a Fujifilm LAS-3000 detector and the membranes were exposed for 5 min.

## Results

### The CiLV-C Encoded Proteins Do Not Suppress Local GFP Silencing Using the 16c RSS System

**FIGURE 1:**
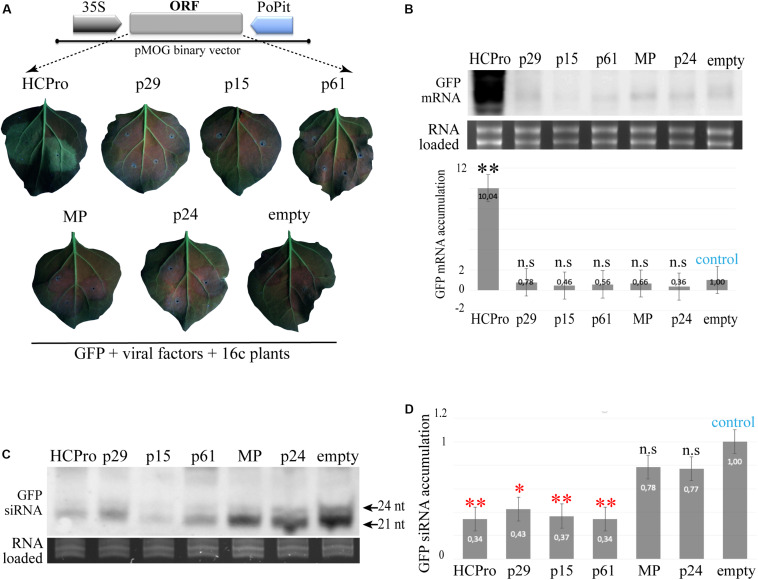
Transient expression of CiLV-C encoded proteins in *Nicotiana benthamiana* 16c and northern blot analysis of the accumulation of GFP derived small RNAs (siRNAs). **(A)** Fluorescent images of *N. benthamiana* 16c leaves co-infiltrated with agrobacterium suspension carrying pMOG_800_ binary vector containing individual CiLV-C ORFs and eGFP used to trigger the silencing of the GFP transgene. The schematic representation corresponds to the expression cassette used for transient expression of the CiLV-C proteins indicated, HCPro and GFP. The ORFs were cloned between CaMV 35S promoter and potato proteinase inhibitor terminator (PoPit). pMOG-HCPro and pMOG empty co-infiltrated with pMOG-GFP were used as positive and negative controls, respectively. The GFP fluorescence was not observed in leaves infiltrated with each of the CiLV-C proteins at 6 days post-infiltration. Three independent experiments were performed, each one included the infiltration of 10 plants per construct. **(B)** Northern blot analysis of GFP mRNA extracted at 4 dpi from the leaves infiltrated with the combination constructs above referred were performed using a dig-riboprobe complementary to the GFP gene. rRNA stained with ethidium bromide indicates equal loading of samples. The graph represents the relative accumulation of mGFP RNAs corresponding to the average of three northern blot from three independent experiments. The value of the averages obtained from the band quantification was normalized in relation to the respective negative control (pMOG-empty). The bands were quantified using the ImageJ version 2.0cr software with ISAC plugin and error bars represent standard deviation. Statistical analysis were done using Student’s *t*-test. Black asterisks (^∗^) indicate significantly increased viral gene accumulation compared to the control. ^∗∗^, *p* < 0.01; n.s, no significant difference. *P*-values were obtained from pairwise comparations between control vs. viral factor. **(C)** Northern blot of GFP-specific siRNA extracted from *N. benthamiana* wt and 16c patches at 4 dpi co-infiltrated with constructs expressing the GFP plus constructs expressing the HCPro (positive control) and p29, p15, p61, MP, and p24 CiLV-C proteins, using a mix of three 50 nt riboprobes complementary to the GFP gene (Martinez-Perez et al., 2019). Negative control (empty) corresponds to patches co-infiltrated with agrobacterium cultures carrying the GFP construct and the empty binary plasmid. Small RNAs of 21 and 24 nt are indicated. rRNA stained with ethidium bromide indicates equal loading of samples. **(D)** The graph represents the relative accumulation of GFP siRNA, referred to the construct carrying the GFP (empty) from three independent experiments. The value of the averages obtained from the band quantification was normalized in relation to the respective negative control (empty). Error bars represent standard deviation. Statistical analyses were done using Student’s *t*-test. Red asterisks (^∗^) indicate significantly decreased viral gene accumulation compared to the control. ^∗∗^, *p* < 0.01; ^∗^, *p* < 0.05; n.s, no significant difference.

*N. benthamiana* 16c plants maintain a visual phenotype of overexpression of the green fluorescent protein (GFP) when the co-transiently expressed tested proteins have RSS activity (Voinnet et al., 1998). To test if any of the CiLV-C proteins have the capacity to suppress local RNA silencing, *N. benthamiana* 16c leaves were simultaneously co-infiltrated with individual *A. tumefaciens* cultures containing the binary vector pMOG-GFP, as gene silencing inducer, and pMOG_800_ constructs containing the viral factors tested for potential RSS. pMOG-HCPro and pMOG-empty constructs were used as positive and negative controls, respectively. When co-infiltrated pMOG-GFP plus pMOG-HCPro, the epidermal leaf cells of 16c plants maintained GFP visual expression under UV illumination after 6 dpi (Figure 1A). In contrast, the leaves co-infiltrated with pMOG-CiLV-C ORFs plus pMOG-GFP, showed decreased GFP expression at 6 dpi, similar to that visualized for the negative control (pMOG-empty) (Figure 1A).

To confirm the results obtained after visual observation, we analyzed the accumulation of GFP mRNA in all combinations tested at 4 dpi. Northern blot analyses revelated a clear positive correlation between the visual GFP expression with abundant mRNA accumulation in leaves expressing the HCPro RSS. For the other tested proteins, the mGFP accumulation was significantly lower (Figure 1B). We also co-expressed, in all possible combinations, groups of two, three or four CiLV-C proteins plus the GFP inducer in 16c plants. However, no increment of the GFP fluorescence was observed in any of the analyzed combination (data not shown).

### Transient Expression of the p29, p15, and p61 Proteins in *N. benthamiana* (wt and 16c Line) Alters siRNA GFP Accumulation

Viral RNA silencing suppressors act in different key components of the RNA silencing, including the block on the production of small RNAs (siRNAs) (Anandalakshmi et al., 1998; Silhavy et al., 2002; Mann et al., 2016). We evaluated whether the CiLV-C proteins were able to interfere in the yield of the small RNA molecules originated from the transiently expressed GFP. To do this, transgenic 16c and wild type *N. benthamiana* leaves were infiltrated with a binary construct carrying the GFP together with constructs carrying a well-known RSS, the HCPro (positive control) or the different CiLV-C ORFs. Northern blot analysis of the accumulation of the GFP-derived siRNAs at 4 dpi revealed that the p29, p15, and p61 reduced significantly the accumulation levels of the small RNAs in both 16 and wt *N. benthamiana* plants (Figure 1D). The northern blot image is representative of all replicates obtained by the infiltration of 16c and wt *N. benthamiana* leaves. Similar reduction was observed with the HCPro positive control. In contrast, a clear signal was detected in the leaves infiltrated with the MP, p24, and pMOG-empty binary vectors (Figure 1C). These results indicate that the p29, p15, and p61 CiLV-C proteins may act as silencing suppressors.

### The Heterologous Expression of the p29, p15, and p61 CiLV-C Proteins Using AMV System Generate Necrotic Lesions on the Inoculated Leaves

**FIGURE 2:**
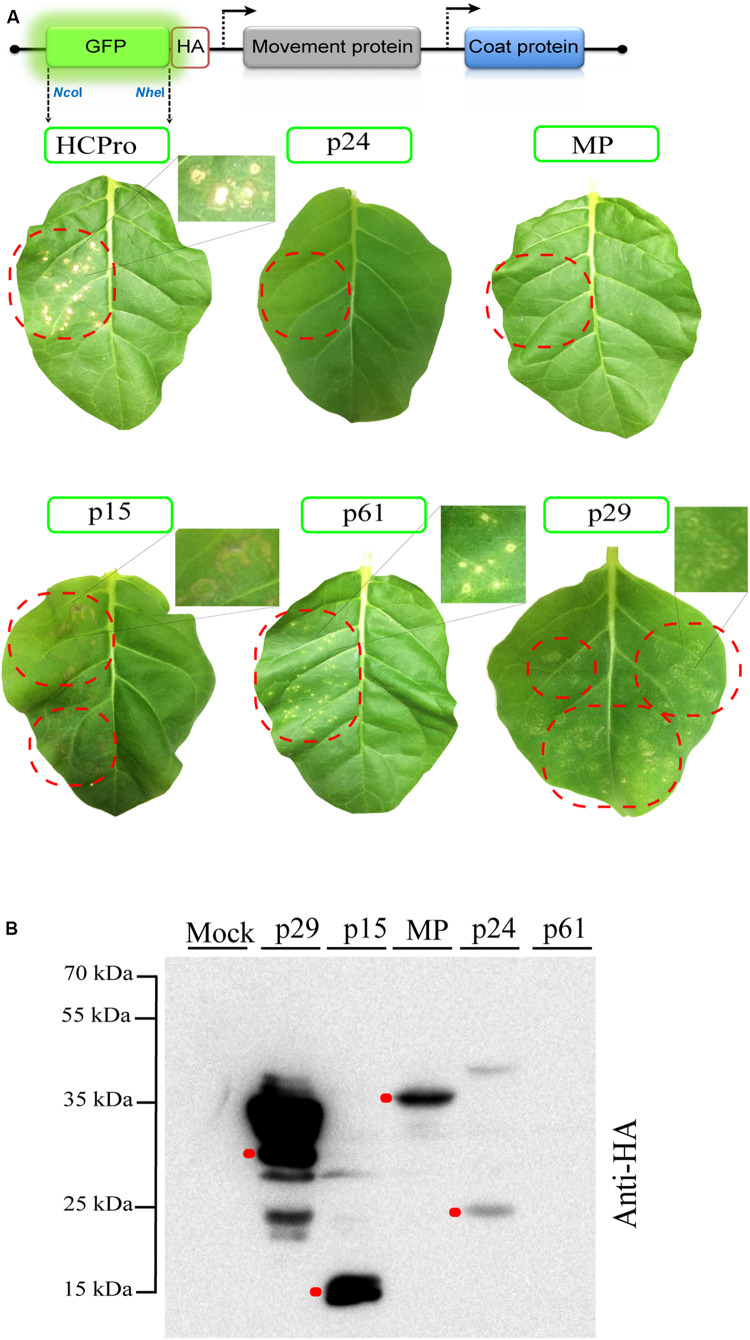
The p29, p15, and p61 proteins trigger necrotic response in P12 *Nicotiana tabacum* leaves. **(A)** Inoculation of P12 leaves with the AMV RNA 3 derivative in which its GFP gene was exchanged by the tobacco etch virus HCPro and CiLV-C ORFs (p29, p15, p61, MP, and P24) fused at their C-terminal with the HA epitope. The schematic representation shows the GFP:HA/MP/CP AMV RNA 3, in which the open reading frames corresponding to the green fluorescent protein (GFP), the movement protein (MP), and the coat protein (CP) are represented by large boxes. Short red box corresponds to the HA epitope, meanwhile arrows represent subgenomic promoters. The *Nco*I and *Nhe*I restriction sites used for insertions of the assayed proteins are indicated. Necrotic response is observed in leaves inoculated with AMV RNA 3 construction expressing the HCPro (positive control), p29, p15, and p61 proteins at 4 days post-inoculation. The dotted red circles indicate the region of viral transcript inoculation. High magnification shows the necrotic lesions. Three independent experiments were performed, each one included the inoculation of three plants per construct; **(B)** Western blot analysis of the accumulation of proteins carrying the HA epitope in P12 leaves at 3 dpi. Red dots indicate the corresponding band for each protein.

Some methods are not always sensitive for identifying viral proteins with suppressive activity. To overcome that, we tested a new and sensitive method based on alfalfa mosaic virus RNA 3 expression vector and transgenic *N. tabacum* p12 plants. For this method, the RSS activity correlates with the appearance of necrotic lesions and increased accumulation of the AMV RNAs (Martinez-Perez et al., 2019). All CiLV-C genes were cloned into the AMV RNA3 carrying the HA epitope fused at their C-termini (Figure 2A). Chimeric AMV RNA 3 transcripts were inoculated on P12 leaves and the phenotypic lesions were monitored for 2 weeks. Chimeric AMV RNA 3 carrying the HCPro and GFP genes were used as positive and negative controls, respectively. P12 plants inoculated with AMV transcripts expressing the p29, p15, and p61 proteins showed necrotic lesions such as those observed for the positive control (Figure 2A). When P12 leaves were inoculated with transcripts expressing the MP and p24 proteins, no symptoms were observed. Absence of symptoms was also observed in the plants inoculated with the negative control (data not shown). No extra symptoms were visualized with longer infection time (30 dpi) in systemic leaves. In order to confirm the expression and stability of the corresponding proteins in absence of any necrotic lesions (Martinez-Perez et al., 2019), extraction of total proteins was performed at 3 dpi from P12 leaves inoculated with the corresponding chimeric AMV constructs. All proteins, except the p61, were detected using a monoclonal anti-HA antibody (Figure 2B). In a previous study, we also failed to detect the p61 protein by Western blot assay (Leastro et al., 2018). To confirm that the necrotic phenotype observed with the AMV construct carrying the p61 protein correlated with its expression, P12 leaves with an RNA 3 variant containing a frameshift version of the p61 gene were inoculated. P12 leaves inoculated with transcripts expressing the p61 with the HA epitope fused either at its N- or C-terminus showed necrotic lesions. However, no symptoms were observed when P12 leaves were inoculated with the p61 frameshift version (Supplementary Figure S1), indicating the correlation between the necrotic phenotype and the p61 expression.

**FIGURE 3:**
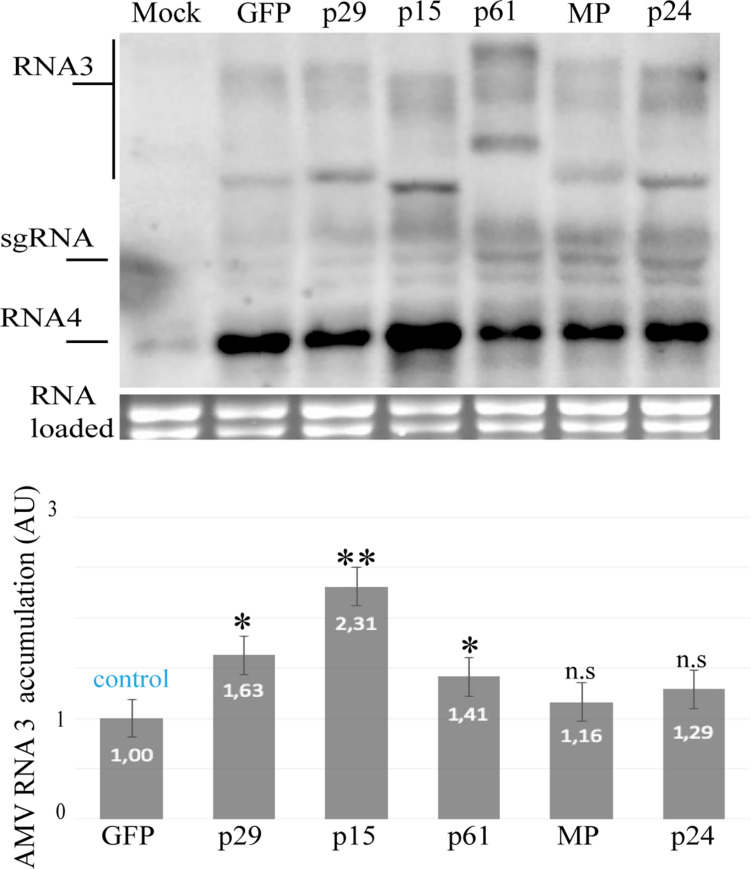
Northern blot analysis of the accumulation of AMV RNA 3 RNA in P12 protoplasts at 16 h post-inoculation, using a dig-riboprobe complementary to the 3’UTR of the AMV. Transcripts correspond to the AMV RNA 3 derivative carrying the p29, p15, p61, MP, and p24 proteins of CiLV-C and GFP protein (negative control). Mock corresponds to the non-infected P12 plant. The localization of RNA 3 and subgenomic RNAs (sgRNA) are indicated. The graph represents the relative accumulation of AMV RNA 3, referred to the construct carrying the GFP from the average of three independent experiments. The value of the averages obtained from the band quantification was normalized in relation to the respective negative control (GFP). The bands were quantified using the ImageJ version 2.0cr software with ISAC plugin and error bars represent standard deviation. Statistical analysis were done using Student’s *t*-test. Black asterisks (^∗^) indicate significantly increased viral accumulation compared to the control. ^∗∗^, *p* < 0.01; ^∗^, *p* < 0.05; n.s, no significant difference. *P*-values were obtained from pairwise comparations between control vs. viral factor.

Next, we evaluated the capacity of the p29, p15, and p61 proteins to increment the AMV accumulation, since the presence of an RSS in AMV RNA 3 incremented its accumulation in P12 protoplasts (Martinez-Perez et al., 2019). For this purpose, the constructs described above were transfected into P12 protoplasts. The quantification of the relative AMV RNA 3 accumulation form northern blot analysis at 16 h post-transfection revealed that the expression of p29, p15, and p61 induces an increment of the AMV RNAs accumulation compared with the GFP control (Figure 3). Taken together, these findings further indicate potential RSS activity for the three CiLV-C encoded proteins.

### The CiLV-C p15 Protein *Trans*-Complements an RSS Defective Turnip Crinkle Virus and Increases the Genomic Viral Accumulation

**FIGURE 4:**
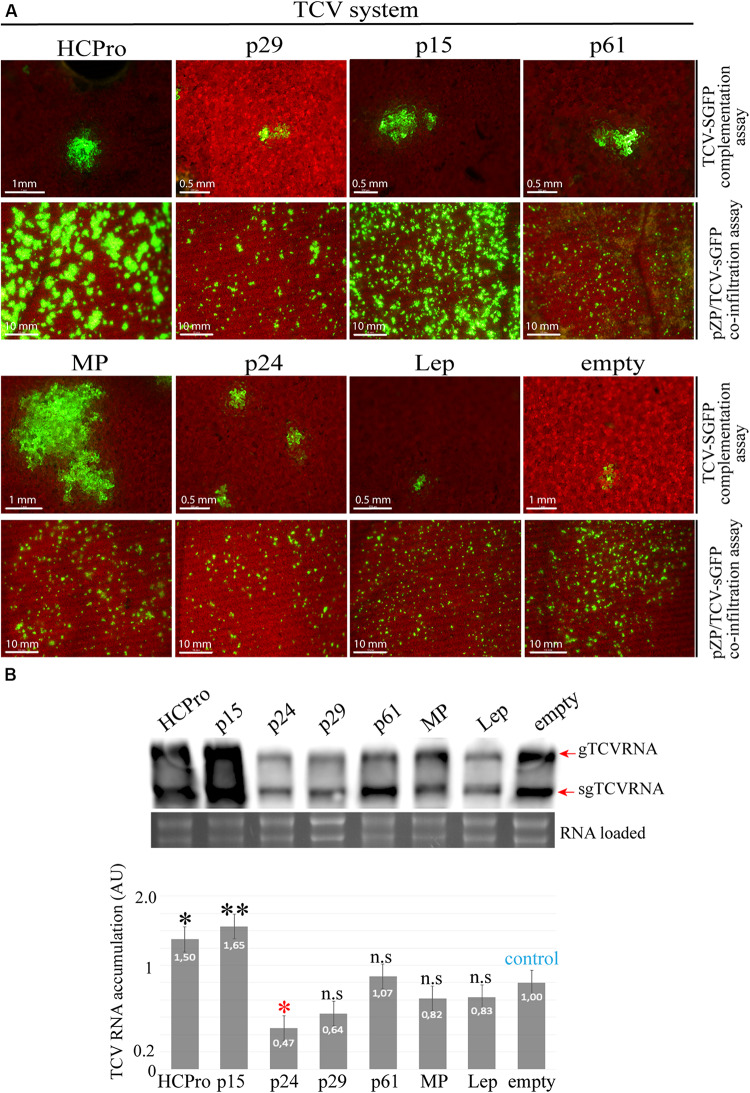
The p15 protein complements the movement of an RSS defective turnip crinkle virus (TCV) mutant. **(A)** TCV assay based on the complementation or increment of fluorescence of the movement-deficiency phenotype of a TCV CP deletion mutant replaced by GFP (TCV-sGFP). For TCV-sGFP complementation assay, three *N. benthamiana* leaves per plant were infiltrated with pMOG_800_ constructs carrying HCPro (positive control), p29, p15, p61, MP, p24, or pMOG-empty and pMOG-Lep as negative controls. Infectious RNA transcripts of the TCV-sGFP were mechanically inoculated 1 day post agroinfiltration. Cell-to-cell movement was evaluated at 3 dpi. For pZP/TCV-sGFP co-infiltration assay, pZP/TCV-sGFP was co-infiltrated with pMOG_800_ empty and Lep (negative controls) or pMOG_800_ carrying HCPro (positive control), p29, p15, p61, MP, and p24 CiLV-C genes. The increment of GFP fluorescence was monitored at 5 dpi. White bars correspond to 500 μm–10 mm. **(B)** Northern blot analysis showing accumulation of TCV-sGFP genomic (gTCV) and subgenomic (sgTCV) RNAs, using a dig-riboprobe complementary to the TCV 3’UTR at 5 dpi. rRNA stained with ethidium bromide indicates equal loading of samples. The graph represents the relative accumulation of gTCV and sgTCV RNAs from three independent experiments. The value of the averages obtained from the band quantification was normalized in relation to the respective negative control (empty). The bands were quantified using ImageJ version 2.0cr software with ISAC plugin and error bars represent standard deviation. Statistical analyses were done using Student’s *t*-test. Black asterisks (^∗^) indicate significantly increased viral accumulation compared to the control, while red asterisks (^∗^) indicate significatively decreased viral accumulation. ^∗∗^, *p* < 0.01; ^∗^, *p* < 0.05; n.s, no significant difference. *P*-values were obtained from pairwise comparations between control vs. viral factor.

To gain additional insights into the RSS functionality for the CiLV-C proteins that show suppression activity, we tested an additional approach for RSS screening based on the functional complementation of a movement-defective TCV mutant (Powers et al., 2008). Leaves infiltrated with the correspondent binary constructions expressing the viral factors at 1 dpi were mechanically inoculated with the movement defective TCV-sGFP transcripts. Foci formation not limited to three or five cells was visualized by expression of the RSS HCPro and CiLV-C p15 and MP proteins (Figure 4A, complementation assay). The remaining CiLV-C proteins did not *trans*-complement the TCV-sGFP movement as observed in the negative controls (Lep and empty) (Figure 4A). To discard that the transport complementation observed for the TCV-sGFP construct could be due to an intrinsic movement function of the assayed protein, the infiltrated leaves were also inoculated with transcripts of a previously characterized TCVΔ92-sGFP construct that has a deletion into the MP ORF, which abolishes the TCV movement but still supports transcription of the subgenomic RNA (Powers et al., 2008). In all cases, except for MP, GFP expression was visualized in individual cells (Supplementary Figure S2), indicating that the ability of p15 and HCPro to complement TCV-sGFP movement was due to an RSS activity rather than an intrinsic movement function, as observed for the CiLV-C MP.

Next, we further tested the RSS activity of the CiLV-C proteins using the PZP-TCV-sGFP co-infiltration assay, whereby the presence of RSS activity is associated with an increase of the GFP signal (Powers et al., 2008; Martinez-Perez et al., 2019). To do this, leaves of *N. benthamiana* were co-infiltrated with individual cultures of *A. tumefaciens* transformed with PZP-TCV-sGFP and pMOG binary constructs carrying the HCPro and all CiLV-C genes. Leaves expressing the p15 protein resulted in a visual increase of GFP signal, in accordance to the HCPro positive control (Figure 4A, co-infiltration assay). In contrast, the other CiLV-C proteins tested did not increment the GFP fluorescent signal (Figure 4A), as observed for the negative controls (Lep and empty). The observation that the CiLV-C MP did not increase the visualized GFP signal further reaffirms that the capability of this protein to restore the transport of the movement defective TCV-sGFP construct correlates with its intrinsic movement function rather than to an RSS activity. Northern blot analyses of the viral RNAs derived from the TCV-sGFP construct showed a significant increase in viral RNAs accumulation in leaves expressing HCPro and p15 proteins, when compared to the negative controls (empty and Lep) (Figure 4B).

### The p29 and p15 Proteins Enhance Pathogenicity of a PVX Infectious Construct, While PVX-p61 Infection Generates a Hypersensitive Response

**FIGURE 5:**
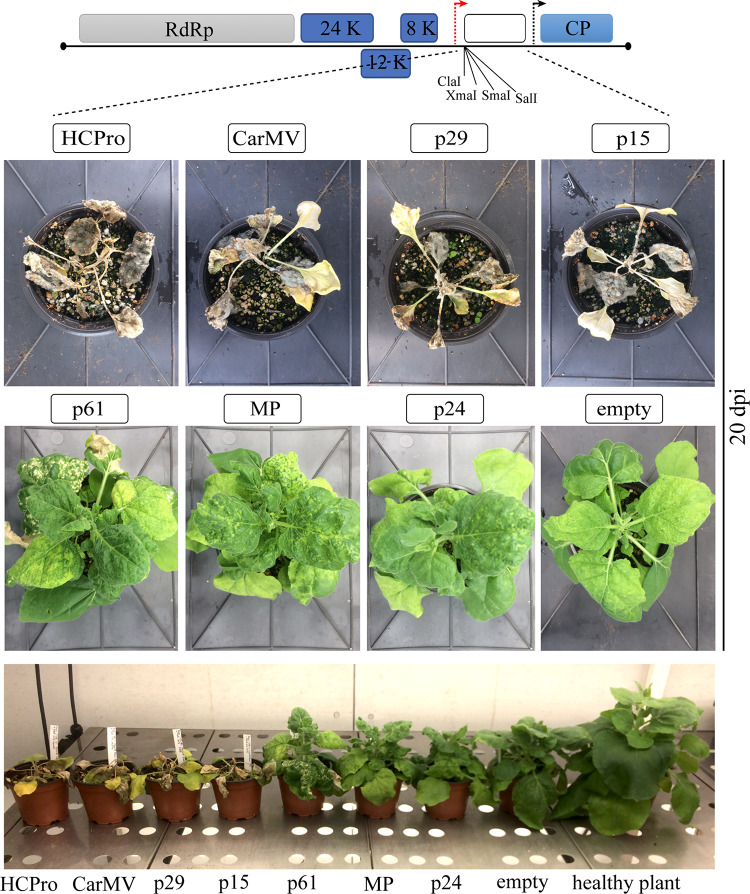
RSS activity of CiLV-C proteins using the potato virus X (PVX) assay. Symptom phenotype of wild type PVX (negative control) and PVX expressing the HCPro, CP CarMV (positive controls), p29, p15, p61, MP, and p24 CiLV-C proteins in *N. benthamiana* plants at 20 dpi. The schematic representation corresponds the pGR107 PVX infectious construct containing *Cla*I, *Xma*I, *Sma*I, and *Sal*I sites downstream of the duplicated PVX CP promoter (red arrow), used for the insertion of the indicated viral ORFs.

**FIGURE 6:**
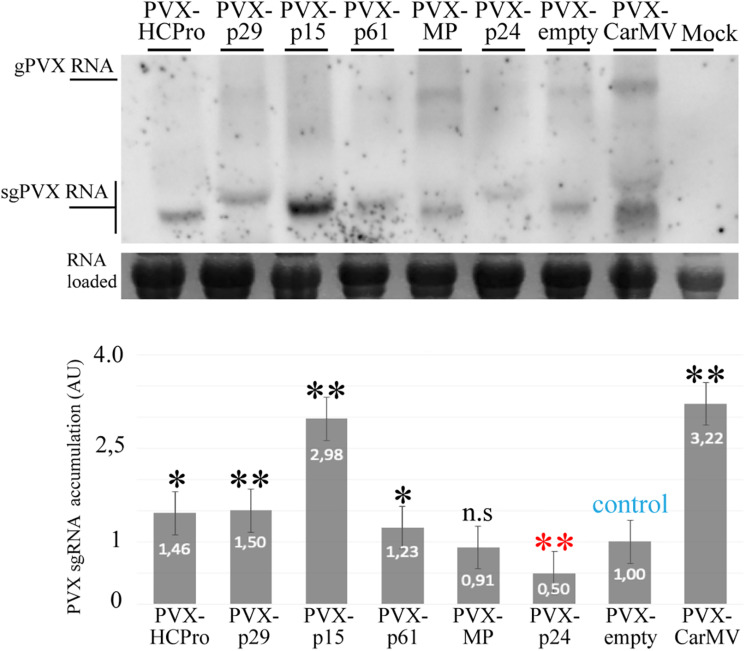
The p29, p15, and p61 expression increase the PVX accumulation. Northern blot analysis showing accumulation of the subgenomic (sgPVX) PVX RNAs using a dig-riboprobe complementary to the 3’ end region of the PVX CP gene at 4 dpi. rRNA stained with ethidium bromide indicates equal loading of samples. Mock corresponds to uninfected plant. The graph represents the relative sgPVX RNA accumulation. The values correspond to the averages of three different experiments in which the data are normalized to the negative control (PVX-empty). The bands were quantified using ImageJ version 2.0cr software with ISAC plugin and error bars represent standard deviation. Statistical analysis were done using Student’s *t*-test. Black asterisks (^∗^) indicate significantly increased viral accumulation compared to the control, while red asterisks (^∗^) indicate significatively decreased viral accumulation. ^∗∗^, *p* < 0.01; ^∗^, *p* < 0.05; n.s, no significant difference. *P*-values were obtained from pairwise comparations between control vs. viral factor.

Using a PVX infectious system, it has been shown that an increase in the severity of the PVX infection suggests the presence of an additional gene with RSS activity (Voinnet et al., 1999; Martinez-Perez et al., 2019). To further test the RSS activity of the CiLV-C proteins we used a recombinant pGR107 PVX construct (Lu et al., 2003) to express the five CiLV-C proteins. *N. benthamiana* leaves were agroinfiltrated with the different PVX derivatives and symptoms development was monitored for 4 weeks. At 4 dpi, all PVX constructs elicited systemic mosaic, mottling, and interveinal chlorosis. At 10 dpi, plants infected with the PVX-p29 and PVX-p15 showed strong symptoms of necrosis in younger leaves, stunted growth, and leaf curling, resulting in complete death at 20 dpi. The same dead phenotype was observed with the positive controls carrying the HCPro (PVX-HCPro) or the CarMV CP (PVX-CP CarMV) (Martinez-Perez et al., 2019; Figure 5). At 3 dpi, the infiltrated and first upper leaves infected with PVX-p61 construct exhibited clear hypersensitive response unable to contain the pathogen and causing systemic mosaic symptoms (Figure 5). For the plants infected with the PVX constructs expressing the MP and p24 CiLV-C proteins, symptoms were similar to those observed for the PVX-empty negative control, but with a little more mosaic (Figure 5). To correlate disease severity with PVX viral accumulation, we performed northern blot analysis at 4 dpi. The quantification of the relative PVX subgenomic RNA accumulation revealed that constructs carrying the p29, p15, and p61 genes and the two positive controls (HCPro and CP CarMV) induced an increment of the PVX RNA accumulation compared with the wild type PVX (Figure 6). These results indicate that p29, p15, and p61 significantly enhanced the pathogenicity of PVX by increasing virus accumulation.

**FIGURE 7:**
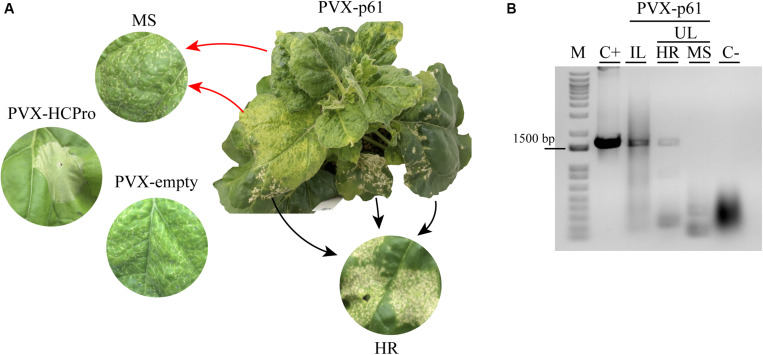
The p61 expression on PVX infection generates a hypersensitive response (HR) phenotype. **(A)** *N. benthamiana* plant infiltrated with pGR107 PVX construct expressing p61 CiLV-C protein. Symptoms of hypersensitive response (HR) and a mosaic of symptoms (MS) are presented in the same upper leave from PVX-p61 infection at 3 dpi. The MS phenotype is also visualized in wild type PVX-empty infection. Necrotic lesions are observed in *N. benthamiana* plants infiltrated with pGR107 PVX construct expressing the RSS HCPro protein. **(B)** RT- PCR analysis to detect the p61 gene (1.650 bp) in inoculated (IL) and upper (UL) leaves of *N. benthamiana* plants inoculated with pGR107-p61 construct, exhibiting HR and MS symptoms. C+, RT-PCR from *N. benthamiama* leaves transiently expressing the p61 (positive control); C-, RT-PCR from uninfected plant sample. The marker band size of 1.500 bp is indicated. M, 1 kb DNA ladder (ThermoFisher Scientific Inc., United States).

On the other hand, the infection derived from PVX-p61 construct presented two different phenotypes in the first upper non-infiltrated leaf, showing a clear hypersensitive response (see HR, Figure 7) together with regions showing mosaic, mottling, and inward leaf curling symptoms (a mosaic of symptoms – MS) (see MS, Figure 7A). The HR phenotype derived from the PVX-p61 construct was clearly distinguished from the necrotic response derived from the PVX-HCPro and PVX-empty variants (Figure 7). In order to correlate the HR phenotype with the presence of the p61 gene, RT-PCR analysis was performed using specific p61 ORF primers from inoculated (IL) and upper (UL) leaves showing either HR or MS phenotypes. The expected p61 amplicon was detected only in the infected tissue showing the HR phenotype (Figure 7B, see lines IL and HR), indicating a direct correlation between this phenotype and the presence of p61 gene. The absence of the p61 amplicon in the infected tissue showing MS phenotype also indicates the low genetic stability of the PVX-p61 construct. For the others PVX derivatives assayed, RT-PCR analysis using specific ORF primers and performed at 14 dpi rendered the expected amplicons in upper non-inoculated leaves, indicating their genetic stability (Supplementary Figure S3).

## Discussion

In this study, we reported that citrus leprosis virus C, the type member of the genus *Cilevirus*, harbors three proteins with RNA silencing suppressor activity, using different assays for the RSS screening (*N. benthamiana* 16c plants, AMV system, siRNA accumulation, RSS defective TCV and PVX pathogenicity system). We observed that three CiLV-C proteins showed RSS activity in almost all evaluated systems but were unable to suppress the local silencing using the 16c assay. These findings show that the method based on *N. benthamiana* 16c is not a foolproof system to screen RSS viral proteins, indicating the continuous need to use other methods to find RSS functionality of unknown viral genes. *Agrobacterium* co-infiltration assay is a relatively quick and easy method to identify RSS proteins, but some RSS are not detected due to their mode of action, sensitivity or lack of intracellular suppression activity (Lu et al., 2004). Regarding the inability of the CiLV-C proteins to suppress the local silencing in 16c plants, two hypotheses can be raised: (1) the p29, p15, and p61 could have a weak suppressive activity at the cellular level or (2) these proteins would not act at the local RNA silencing process, performing their blocking activity in another step of the silencing defense machinery. Unfortunately, it was not possible to test the ability of the CiLV-C proteins to prevent the systemic spread of the GFP transgene silencing signal in 16c plants and, therefore, further experiments are needed to address this question. It is interesting to note that although the p29, p15, and p61 CiLV-C proteins were unable to induce the local RSS in 16c plants, their expression in wild type or transgenic *N. benthamiana* 16c, reduced considerably the accumulation of small RNAs, indicating that the “patch” technique is more efficient by analyzing the small RNAs accumulation derived from the reporter gene rather than to perform the screening only by detecting the fluorescent signal. Collectively, these data suggest that these viral proteins interfere in a step after dsRNA production, whereby the decrease of siRNA accumulation can be explained by a possible binding of the proteins to 21 and 24 nt siRNAs. Revealing whether the p29, p15, and p61 have affinity for 21 or 24 nt siRNAs would help clarify this issue.

In contrast to the negative results obtained with the “patch” technique based on the 16c plants, the *cis*-expression of the p29, p15, and p61 induced a phenotype of necrotic lesions in AMV infection context, increasing the viral RNA accumulation. Also, these three cilevirus proteins caused a substantial reduction of siRNA accumulation when transiently expressed in leaf patches of 16c and wild type *N. benthamiana* plants and enhanced the PVX pathogenicity, incrementing its viral RNA accumulation. Taken together, these data indicate that the p29, p15, and p61 are silencing suppressors of CiLV-C. Examples of multiple-components RNA silencing suppression viral mechanisms have been reported for criniviruses, closteroviruses, geminiviruses, emaraviruses, and potyviruses (Lu et al., 2004; Vanitharani et al., 2004; Canizares et al., 2008; Gupta et al., 2018; Rodamilans et al., 2018). This plurality of proteins with silencing suppressor activity from a single viral entity may indicate a constant viral adaptation to counteract the plant defense mechanism.

In our previous study, we showed that p15 is localized into the nucleus of *N. benthamiana* epidermal cells with unknown function (Leastro et al., 2018). Here, we clearly identified the p15 RSS activity using four different methods. The nuclear localization of p15 strongly suggests that this protein accesses the nucleus possibly to block RNA silencing. This same feature has been demonstrated for other RSS viral proteins, i.e., the 2b proteins of cucumber mosaic virus (CMV), which nuclear localization is a prerequisite for an efficient suppression of PTGS (Lucy et al., 2000). In arabidopsis, the siRNA processing, which encompasses RdRP activity, Dicer processing and Ago mediated target cleavage are all intimately linked in the nucleus (Castel and Martienssen, 2013).

The suppression of RNA silencing activity of unknown viral genes has been identified from screening based on functional complementation of defective viral mutants (Chiba et al., 2006; Powers et al., 2008; Martinez-Perez et al., 2019). The p15 was able to rescue the cell-to-cell movement of the RSS defective TCV and to increase the GFP signal, further supporting that p15 is a silencing suppressor of CiLV-C. In contrast, the p29 and p61 CiLV-C proteins were incapable to rescue the viral movement and to increment the GFP expression in this system. The PVX p25, a viral protein that has been previously reported incapable of suppressing local silencing, acting exclusively in steps associated with intercellular suppression silencing (Voinnet et al., 2000) also was unable to rescue the movement of the RSS TCV defective construct (Powers et al., 2008). On the other hand, p29 and p61 RSS activities were identified from small RNA accumulation analyses and with the AMV and PVX system. Taken together, these data suggest that these proteins may affect the RNAi pathway (Mallory et al., 2002) differently than p15. The TCV system was unable to identify RSS activity for those suppressors that act blocking the spread of silencing signal (Powers et al., 2008), suggesting that p15 probably acts at the level of local silencing, however, it seems to be a weak local suppressor, given its inability to suppress local silencing in 16c plants.

We recently proved that cilevirus movement protein (p32) is efficiently able to generate viral cell-to-cell and long-distance spread in heterologous systems (Leastro et al., unpublished), indicating that limitation of the cileviruses to systemically infect their hosts (Freitas-Astua et al., 2018) is not due to a functional restriction in their MPs. In this sense, although speculative, we infer that the putative weak suppression activity observed for the CiLV-C RSS proteins could explain, at least in part, the systemic movement impairment, since the RNA silencing could hinder the vascular transport of viruses, which inhibits viral entry into the phloem (Yelina et al., 2002; Vuorinen et al., 2011). Our above suggestion is reinforced from RSS studies with the proteins of orchid fleck virus (OFV-citrus), a dichorhavirus also involved in the citrus leprosis complex (Roy et al., 2013). As demonstrated for CiLV-C, the identified RSS protein encoded by OFV also shows inability to suppress local RNA silencing in 16c plants (Leastro et al., unpublished) and dichorhaviruses also have limitations to systemically infect their hosts. The observation that the incapacity of cileviruses to infect their hosts is not limited to one host, but to more than 50 different natural and experimental host species (Garita et al., 2014) opens the possibility that a general mechanism (e.g., plant RNA silencing) could be responsible for impairing the long movement of these citrus leprosis-associated viruses. However, further experiments are needed to address this question.

The p29 and p15 significantly enhanced the PVX accumulation and symptoms severity, including death of tobacco plants. Similar results were previously observed in plants agroinfiltrated with PVX recombinants expressing several viral suppressor proteins (Brigneti et al., 1998; Pfeffer et al., 2002; Thomas et al., 2003; Delgadillo et al., 2004; Canizares et al., 2008; Gupta et al., 2018; Martinez-Perez et al., 2019). We observed that the p61 also enhanced the PVX accumulation, although symptom severity was not maintained over time due to the instability of the PVX-p61 construct. However, during the onset of the PVX-p61 infection, a clear hypersensitive necrosis was observed similar to that visualized during the transient expression of the p61 protein (Leastro et al., 2018). A hypersensitive-like response has been suggested as an outcome of CiLV-C infection in arabidopsis plants (Arena et al., 2016) and our data further reinforces the idea that, together with the RSS activity, p61 could also activate a plant defense response mechanism, acting as a pathogenicity determinant. On this context, recently Arena et al. (unpublished) also showed that the expression of p61 protein triggers HR and furthermore mimics plant responses to viral infection.

Collectively, our findings establish that p29, p15, and p61 proteins possess RSS activity. Thus, we elucidated at least part of the functionality of the p15 protein, and provided some additional functions for the capsid protein (p29) and the putative glycoprotein (p61) of the citrus leprosis virus C.

## Data Availability Statement

All datasets generated for this study are included in the article/Supplementary Material.

## Author Contributions

ML, DC, and JS-N conceived and designed the experiments, analyzed, and interpreted the data. ML and DC performed the experiments. ML, JF-A, EK, VP, and JS-N contributed reagents, materials, and tools. ML wrote the original draft preparation. ML, JF-A, VP, and JS-N revised and edited the manuscript.

## Conflict of Interest

The authors declare that the research was conducted in the absence of any commercial or financial relationships that could be construed as a potential conflict of interest.

## Supplementary Material

FIGURE S1 Confirmation of p61 expression on AMV infection using a frameshift version of p61 gene. *Nicotiana tabacum* P12 leaves inoculated with three variants of the AMV RNA 3 construct, which express the p61 ORF with the HA epitope fused at its N- (HA:p61) or C- (p61:HA) termini or with a frameshift mutation in the 5’terminal region (p61fs). The schematic representation shows the GFP:HA/MP/CP AMV RNA 3, in which the open reading frames correspond to the green fluorescent protein (GFP), the movement protein (MP) and the coat protein (CP) are represented by large boxes. In the assayed AMV constructs the GFP gene is changed with the different p61 indicated versions. Necrotic response observed at 4 dpi. Arrows represent subgenomic promoters and doted circles delimited the inoculated leaf area. Three independent experiments were performed, each one included the infiltration of three leaves per plants and three plants per construct.

FIGURE S2 The CiLV-C p15 does not complement movement of the TCVΔ92-sGFP mutant. *N. benthamiana* leave were infiltrated with pMOG_800_ expressing the HCPro and CiLV-C MP and p15 proteins. Infectious RNA transcript of the TCVΔ92-sGFP construct were mechanically inoculated one day post agroinfiltration. Cell-to-cell movement evaluated at 3 dpi. White bars correspond to 0.5–2 mm.

FIGURE S3 RT- PCR analysis to confirm the genetic stability of the PVX constructs expressing the CiLV-C p29 (792 bp), p15 (393 bp), p61 (1614 bp), MP (894 bp) and p24 (645 bp) proteins. RT-PCR performed from upper leaves of *N. benthamiana* plants infected at 14 dpi using specific primers for each gene. C+, RT-PCR from *N. benthamiama* leaves transiently expressing the HCPro (positive control); C−, RT-PCR from uninfected plant sample. The marker band size of 300, 650, 1,000, and 1,500 bp are indicated. M, 1 kb DNA ladder.
