# Supplementary figures and images for: Citrus Leprosis Virus C Encodes Three Proteins With Gene Silencing Suppression Activity
Source: Front Microbiol. 2020 Jun 9;11:1231. doi: 10.3389/fmicb.2020.01231 (PMC7325951; doi:10.3389/fmicb.2020.01231)

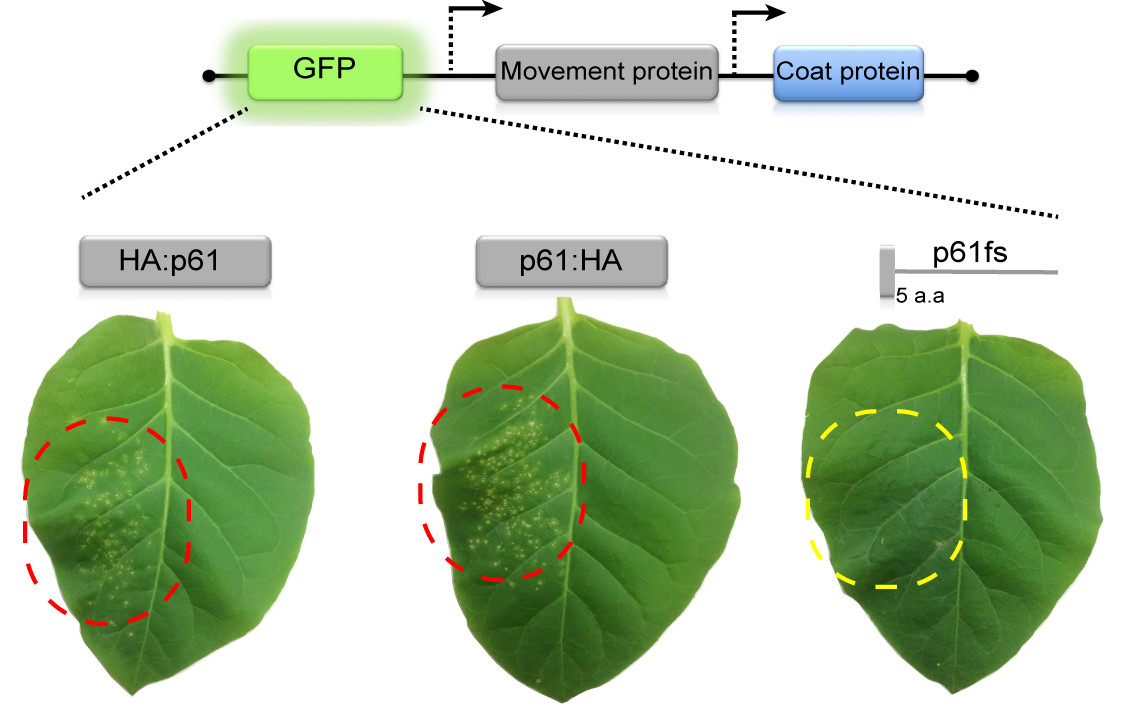

Supplement: FIGURE S1 — Confirmation of p61 expression on AMV infection using a frameshift version of p61 gene. Nicotiana tabacum P12 leaves inoculated with three variants of the AMV RNA 3 construct, which express the p61 ORF with the HA epitope fused at its N- (HA:p61) or C- (p61:HA) termini or with a frameshift mutation in the 5’terminal region (p61fs). The schematic representation shows the GFP:HA/MP/CP AMV RNA 3, in which the open reading frames correspond to the green fluorescent protein (GFP), the movement protein (MP) and the coat protein (CP) are represented by large boxes. In the assayed AMV constructs the GFP gene is changed with the different p61 indicated versions. Necrotic response observed at 4 dpi. Arrows represent subgenomic promoters and doted circles delimited the inoculated leaf area. Three independent experiments were performed, each one included the infiltration of three leaves per plants and three plants per construct. [file Image_1.TIF]

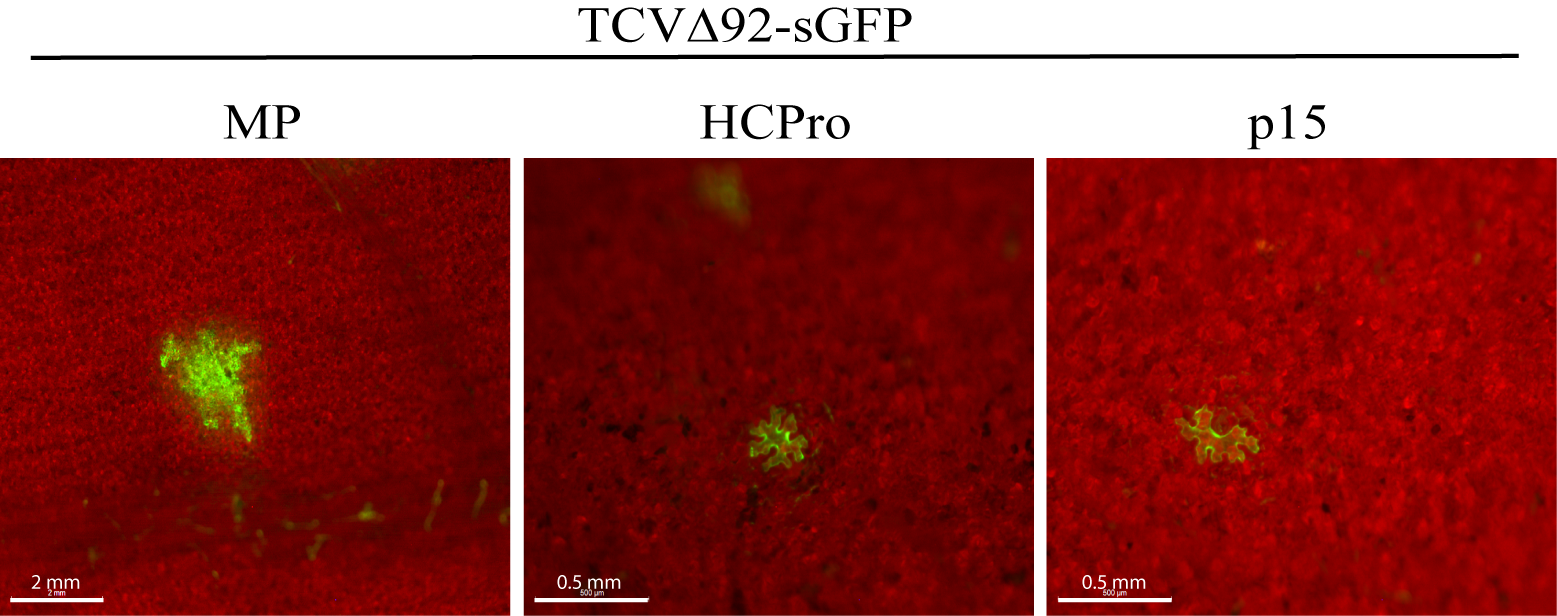

Supplement: FIGURE S2 — The CiLV-C p15 does not complement movement of the TCVΔ92-sGFP mutant. N. benthamiana leave were infiltrated with pMOG800 expressing the HCPro and CiLV-C MP and p15 proteins. Infectious RNA transcript of the TCVΔ92-sGFP construct were mechanically inoculated one day post agroinfiltration. Cell-to-cell movement evaluated at 3 dpi. White bars correspond to 0.5–2 mm. [file Image_2.TIF]

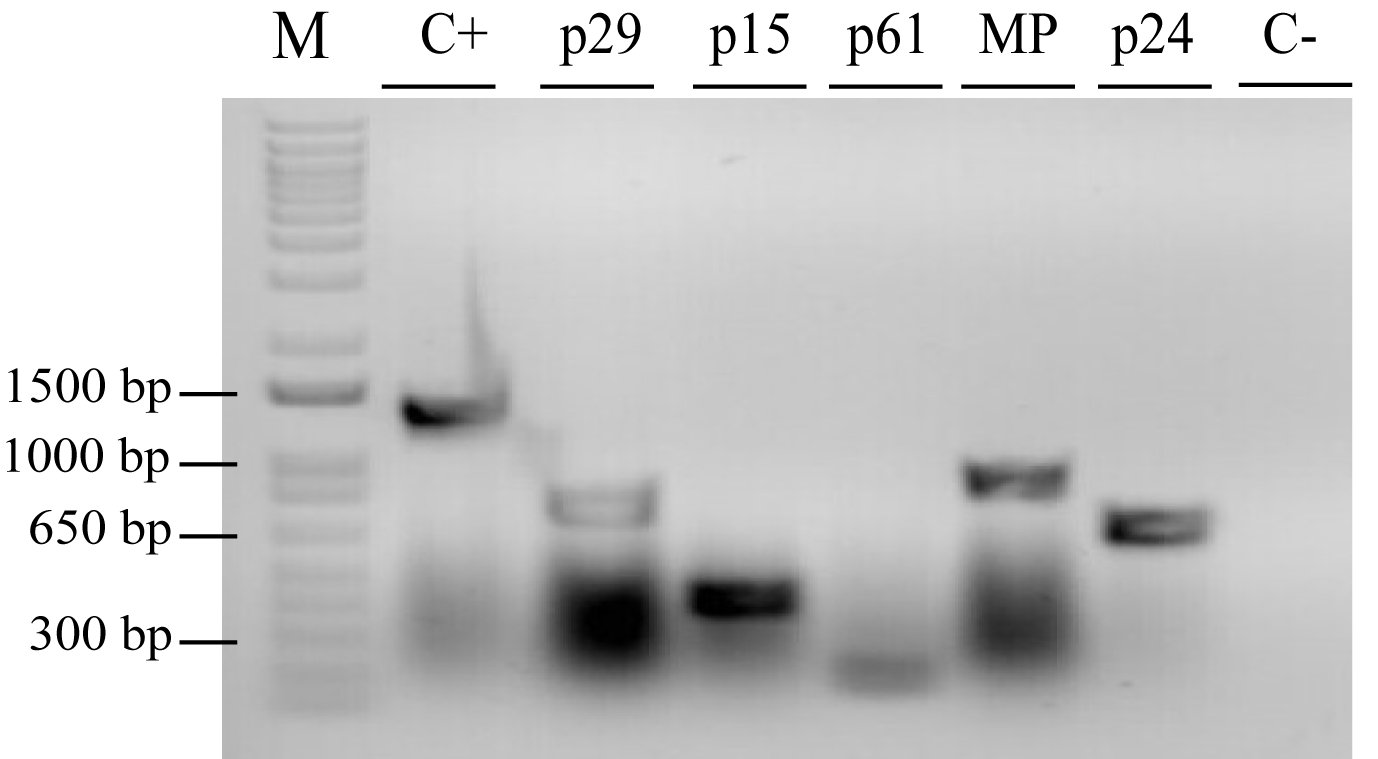

Supplement: FIGURE S3 — RT- PCR analysis to confirm the genetic stability of the PVX constructs expressing the CiLV-C p29 (792 bp), p15 (393 bp), p61 (1614 bp), MP (894 bp) and p24 (645 bp) proteins. RT-PCR performed from upper leaves of N. benthamiana plants infected at 14 dpi using specific primers for each gene. C+, RT-PCR from N. benthamiama leaves transiently expressing the HCPro (positive control); C−, RT-PCR from uninfected plant sample. The marker band size of 300, 650, 1,000, and 1,500 bp are indicated. M, 1 kb DNA ladder. [file Image_3.TIF]
